# Halotolerant Rhizobacterial Strains Mitigate the Adverse Effects of NaCl Stress in Soybean Seedlings

**DOI:** 10.1155/2019/9530963

**Published:** 2019-10-20

**Authors:** Muhammad Aaqil Khan, Sajjad Asaf, Abdul Latif Khan, Arjun Adhikari, Rahmatullah Jan, Sajid Ali, Muhammad Imran, Kyung-Min Kim, In-Jung Lee

**Affiliations:** ^1^School of Applied Biosciences, Kyungpook National University, Daegu 41566, Republic of Korea; ^2^Natural and Medical Sciences Research Center, University of Nizwa, Nizwa 616, Oman; ^3^Research Institute for Dok-do and Ulleung-do Island, Kyungpook National University, Daegu, Republic of Korea

## Abstract

**Background:**

Salinity is one of the major abiotic constraints that hinder health and quality of crops. Conversely, halotolerant plant growth-promoting rhizospheric (PGPR) bacteria are considered biologically safe for alleviating salinity stress.

**Results:**

We isolated halotolerant PGPR strains from the rhizospheric soil of *Artemisia princeps, Chenopodium ficifolium, Echinochloa crus-galli,* and *Oenothera biennis* plants; overall, 126 strains were isolated. The plant growth-promoting traits of these isolates were studied by inoculating them with the soil used to grow soybean plants under normal and salt stress (NaCl; 200 mM) conditions. The isolates identified as positive for growth-promoting activities were subjected to molecular identification. Out of 126 isolates, five strains—*Arthrobacter woluwensis* (AK1), *Microbacterium oxydans* (AK2), *Arthrobacter aurescens* (AK3), *Bacillus megaterium* (AK4), and *Bacillus aryabhattai* (AK5)—were identified to be highly tolerant to salt stress and demonstrated several plant growth-promoting traits like increased production of indole-3-acetic acid (IAA), gibberellin (GA), and siderophores and increased phosphate solubilization. These strains were inoculated in the soil of soybean plants grown under salt stress (NaCl; 200 mM) and various physiological and morphological parameters of plants were studied. The results showed that the microbial inoculation elevated the antioxidant (SOD and GSH) level and K^+^ uptake and reduced the Na^+^ ion concentration. Moreover, inoculation of these microbes significantly lowered the ABA level and increased plant growth attributes and chlorophyll content in soybean plants under 200 mM NaCl stress. The salt-tolerant gene *GmST1* was highly expressed with the highest expression of 42.85% in AK1-treated plants, whereas the lowest expression observed was 13.46% in AK5-treated plants. Similarly, expression of the IAA regulating gene *GmLAX3* was highly depleted in salt-stressed plants by 38.92%, which was upregulated from 11.26% to 43.13% upon inoculation with the microorganism.

**Conclusion:**

Our results showed that the salt stress-resistant microorganism used in these experiments could be a potential biofertilizer to mitigate the detrimental effects of salt stress in plants via regulation of phytohormones and gene expression.

## 1. Introduction

Salinization is a devastating environmental stress factor that greatly limits plant growth and productivity [1]. About 800 million hectares of earth's land has been recognized as having a high salinity level globally [2]; this includes >15% land of the arid and semiarid regions and approximately 40% of the world's irrigated land [3]. Recently, Etesami and beattie [4] reported that salinity stress decreases the productivity of important cereal crops like wheat, maize, rice, and barley by up to 70% in different areas. Both direct and indirect effects of soil salinity inhibit the growth and productivity of crops. The direct adverse effects of salinity stress on plants include osmotic stress induced on root surface, compromised water acquisition, and toxic ion (e. g., Na^+^) accumulation in plant cells, which lead to nutrient deficiency and growth retardation of different plants [5–10]. Furthermore, even indirect effects of salinity stress greatly hamper the activities of various beneficial microbes present in the rhizosphere and decrease organic matter accumulation [11].

Salinity is a major limitation to the growth of several different agriculture crops all over the world, and various approaches have been applied to mitigate the adverse effects of salinity stress. Recently, various eco-friendly techniques were proposed and applied to mitigate the harmful effects of salt stress on various crops, including soybean. To achieve such an environment-friendly solution, it is necessary to isolate and identify plant growth-promoting microbes that can enhance soybean growth during salt stress. Salinity stress induces different biochemical and molecular effects in plants through reactive oxygen species (ROS) production, which could be manifested in the form of anatomical and physiological changes [12]. High concentrations of ROS are extremely harmful and lead to unwanted effects on plants at a cellular level, such as chlorophyll degradation, cell membrane oxidation, and the eventual cell death [13]. The detoxification of high ROS levels in plants is achieved by the development of different antioxidant enzymes. In connection to this, the well-known enzymes, such as superoxide dismutase (SOD) and reduced glutathione (GSH), have been reported for their ability to eradicate free radicals produced during salinity stress on the plants [13]. Various studies have shown a marked increase in the antioxidant defense system, adaptability, and survival of plants in a stress-inducing environment [14]. Higher concentration of salt (NaCl) causes ionic imbalance, which leads to osmotic stress and ionic toxicity [15]. Since Na^+^ and K^+^ play a major role in plant physiology during salt stress conditions, expulsion or efflux of Na^+^ and influx of K^+^ are the most important strategies to alleviate salinity stress in plants. The influx of sodium ion concentration affects low-affinity potassium uptake by the roots and causes water deficiency, thus causing an osmotic effect that has a devastating effect on plant growth, such as ceasing photosynthesis, low stomatal conductance, unusual transpiration rate, and reduced chlorophyll concentration [16].

Among crops, soybean is one of the most important and staple legume crop providing high oil (18%) and protein (38%) contents. It is moderately tolerant to salinity stress [17, 18], with 20%–50% reduction in the production under high salinity stress [19]. Thus, salinity stress significantly affects all the developmental stages from seed germination to seed yield [20]. During soybean development, salt stress reduces plant biomass, chlorophyll content, the number of internodes, the number of pods, and seed quality [20–22]. The availability of the whole genome sequence of soybean improved our understanding of the basic mechanisms of how salinity affects gene expression and regulation [20]. Salt stress-tolerance genes, such as auxin-resistant 1 (*GmLAX*) and soybean salt tolerance 1 (*GmST1*), are greatly involved in ABA signaling and mitigating ROS stress; these genes are also known as salt stress-responsive genes [17, 20, 23, 24]. *Gm-ST1* encodes cation/proton exchanger family members and is responsible for conferring NaCl stress tolerance to plants at different stages, such as seedling and adult stage [17, 20]. Additionally, the gene expression of *Gm-ST1* transgenic lines also increased ABA sensitivity and decreased ROS production under prolonged salt stress conditions [17, 25]. Similarly, *GmLAX*, encoding a putative auxin influx carrier, is also involved in abiotic stress responses and plays a very prominent role in different aspects of plant growth and development; for example, it has an important role in vascular development and regulation of auxin uptake or transport [26]. Researchers have already reported that *GmLAX* also plays an important role in regulation of vascular development, xylem differentiation, and regulation of lateral root growth [27–29].

A number of approaches were used to address the negative impact of salinity stress, including gypsum application, recombinant DNA technology approach, and traditional breeding, because planting salt-tolerant crop varieties had limited successes despite significant efforts [18, 30–33]. An alternative strategy to alleviate salinity stress is the application of halotolerant bacteria that enhance crop growth under salt stress conditions [30, 34]. The most commonly used halotolerant PGPR bacteria are *Acinetobacter*, *Azotobacter*, *Bacillus* sp., *Serratia* sp., *Pseudomonas* sp., and *Rhizobium* sp., which enhance plant growth by nitrogen fixation, promote inorganic phosphate solubilization, and promote siderophore and phytohormone production [35–39]. Similarly, a number of different reports have revealed that halotolerant microbes enhance the growth and development of various crops (rice, wheat, maize, tomato, soybean, lettuce, cotton, pepper, and canola) under both normal and salinity stress conditions [1, 12, 25, 40–43]. Thus, in the present study, we aimed to isolate and characterize halotolerant PGPR bacteria for mitigating salinity stress damages in the growth and development of soybean plants. We also elucidated the role of K^+^ and Na^+^ translocation, stress hormone (ABA), and antioxidants (GSH and SOD). Additionally, the expression of the candidate salt stress-responsive genes *GmLAX3* and *GmST1* was evaluated in soybean plants.

## 2. Materials and Methods

### 2.1. Isolation and Screening of PGPR Bacteria and Their Capabilities

During sampling, we isolated a number of different bacterial strains from the near vicinity of different plants inhabiting sand dunes at Pohang beach with a latitude of 36°7′56.2″N and a longitude of 129°23′55.1″E, Republic of Korea. The detailed method of Khan et al. [25] was followed for the isolation of PGP rhizospheric bacteria from the rhizospheric soil (1 g) of *O. biennis* L., *A. princeps* Pamp, *C. ficifolium* Smith, and *E. crus-galli*. The plant roots were excised along with rhizospheric soil, placed into 10 mL of sterile 0.9% NaCl solution, and vortexed for 10 min to detach the associated rhizospheric bacteria. After serial dilutions (10^−1^ until 10^−9^), the samples were inoculated on LB agar medium (tryptone, 10 g; yeast extract, 5 g; NaCl, 10 g; and agar, 1.5%; pH 7.0) and incubated (28°C) till the appearance of bacterial colonies; then, they were observed for the morphological characteristics.

Before bioassay assessment and molecular identification, the pure culture of selected isolates was screened for phosphate solubilization and siderophore and indole acetic acid (IAA) production. For siderophore production, the detailed methods of Khan et al. and Louden et al. [25, 44] were followed using chrome azurol S blue agar media. Each bacterial isolate was spot inoculated and incubated at 28°C and then analyzed for the appearance of orange halos in contrast to the blue background for five consecutive days. The detailed method of Katznelson and Bose [45] was assessed for phosphate solubilization using trypticase soy agar (TSA) medium supplemented with Ca_3_(PO_4_)_2_. Each bacterial isolate was spot inoculated and incubated at 28°C for 5 days until the formation of transparent “halos” around each colony. The method of Patten and Glickk [46] was used to detect the bacterial IAA in culture broth. The supernatant (1 mL) of each bacterial isolate was added in 1 mL of Salkowski reagent (50 mL of 35% HClO_4_ and 1 mL of 0.5 M FeCl_3_) for 30 min in dark condition. Visual assessment of the change in pink color indicated IAA production.

### 2.2. Bioassay Assessment and Molecular Identification of Bacterial Isolates

Based on multiple PGP traits, a total of seven isolates were capable of multiple traits in different media. Hence, the selected isolates were subjected to further analysis on *Waito-C* (GA-deficient) rice seedlings [47, 48]. Seeds of *Waito-C* (stored in desiccators at 4°C) were surface sterilized by soaking in 10 mL of 75% ethanol for 2 min, followed by 1% sodium hypochlorite (NaClO) treatment for 1 min. Then, they were thoroughly washed five times with sterilized distilled water. Sterilized seeds were treated for 24 h with bacterial isolates (10^9^ CFU/mL) in a shaking incubator. Conversely, autoclaved double-distilled water was used for untreated seeds used as control. The test seeds were grown over an autoclaved filter paper, which were soaked in 1.5 mL of Hoagland solution for 14 consecutive days under controlled environmental conditions (14 h/10 h light/dark cycle; temperature, 28°C/24°C) at a relative humidity of approximately 70% and light intensity of 250 *μ*mol m^−2^s^−1^. Bacterial isolates that enhanced the growth and development of rice seedlings were evaluated for further experiments. The selected isolates after screening experiments on *Waito-C* rice were identified through 16S rDNA gene amplification using 27F (5′-AGA GTT TGA TC(C/A) TGG CTC AG-3′) and 1492R (5′-CGG (T/C)TA CCT TGT TAC GAC TT-3′) primers, and BLAST search tool of NCBI (http://blast.ncbi.nlm.nih.gov) was used for sequence alignment. Similarly, GenBank (database/EzTaxon) was used to determine the nucleotide sequence homology of the targeted bacterial isolate, and MEGA v. 6.1 was used for phylogenetic analysis by constructing a neighbor joining (NJ) using 16S rDNA gene sequences from selected and related strains [49].

### 2.3. Quantification of *In Vitro* IAA and GAs in Bacterial Culture through GCMS-SIM

The bacterial strains were grown in LB media (10 g tryptone, 5°g yeast extract, and 10°g NaCl with a pH of 7.0) for 72 h, centrifuged at 5000*g* for 10 min, and filtered through a 45 *μ*m filter. The isolated culture filtrate (CF) was analyzed for different types of IAAs and GAs through gas chromatography-mass spectrometry under selected ion monitoring mode (GC/MS SIM). The detailed method of Ullah et al. [50] was used for IAA analysis. To measure IAA concentration in the broth, the peak areas of IAA were compared to the known standard using GC/MS SIM. For the extraction and quantification of GA contents, the detailed method of Khan et al. [51, 52] was followed. GA internal standards ((17, 17-2H_2_) GA_1_, GA_3_, GA_4_, GA_7_, GA_8_, GA_9_, GA_12_, GA_19_, GA_20_, GA_24_, and GA_36_) were added to CF before performing column chromatography. All extracts were passed through a C18 column (90–130 *μ*m; Alltech, USA) to obtain different fractions. For each type of GA, 1 *μ*L aliquot was injected into the GC/MS column. The amounts of GAs (GA_1_, GA_3_, GA_4_, GA_7_, GA_8_, GA_9_, GA_12_, GA_19_, GA_20_, GA_24_, and GA_36_) in CF were calculated from the peak-area ratios, and retention time was determined using hydrocarbon standards.

### 2.4. Quantification of Organic Acids

The organic acid was quantified using the method of Kang et al. [53] and Khan et al. [47]. Briefly, the bacterial culture in the LB medium was centrifuged at 5000*g* for 20 min. The culture supernatant was adsorbed using Sep-Pak C18 cartridge (Waters, Milford, MA, USA) and filtered through a 0.45 *μ*m cellulose acetate membrane filter. The samples were analyzed through high-performance liquid chromatography (HPLC) (Waters 600, Milford, MA, USA) using a PL Hi-Plex H column (7.7 × 300 mm, Waters Co., Milford, MA, USA), detector refractive index (RI) (Waters 410, Milford, MA, USA), and 5 mM H_2_SO_4_ as the solvent in distilled water. The flow rate was set to 0.6 mL min^−1^ with 65°C oven temperature and 20 *μ*L injection volume.

### 2.5. Pot Experiment

In the present study, we used soybean seeds of cv. Pungsannamul were collected from Kyungpook National University's Genetic Resource Centre, Republic of Korea. All the seeds were surface sterilized with 2.5% sodium hypochlorite for 20 min, followed by treatment with 70% ethanol for 30 s; then, they were washed three times with deionized double-distilled water and subjected to germination. Uniformly germinated seedlings were selected and transferred to sterilized pots filled with autoclaved soil. We used specific soil prepared by Punong Co., Ltd., Korea, which comprised perlite (11%), cocopeat (68%), zeolite (8%), NO_3_^−^ (∼0.205 mg g^−1^), NH^4+^ (∼0.09 mg g^−1^), P_2_O_5_ (∼0.35 mg g^−1^), and K_2_O (∼0.1 mg g^−1^). The selected seedlings were grown in a growth chamber: 14 h/10 h light/dark cycle; temperature, 28°C/24°C; relative humidity, 60%–70%; and light intensity, 1000 *μ*E m^−2^ s^−1^ from sodium lamps. The seedlings were irrigated with autoclaved distilled water as required. There were seven treatments in this experiment, including control-untreated rice seedlings, control-NaCl (treated with 150°mM NaCl), and five treatments with five different bacterial isolates. However, the seedlings treated with NaCl were also inoculated with bacterial isolates for evaluating their combined effects on plant growth and development. After stress treatment, the plants were directly subjected to liquid nitrogen and stored at −80°C for further analysis. Finally, different plant growth attributes were recorded, and for chlorophyll content measurement, Minolta SPAD-502 (Konica Minolta, Japan) was used. Fresh samples were used for gene expression analysis, and lyophilized samples were used for other analyses.

### 2.6. Molecular Analysis to Understand Transcript Involved in Salinity Stress

The RNA was extracted using the protocol described by Chan et al. [54]. The leaf tissues of soybean seedlings were treated with liquid nitrogen to grind them to form a fine powder of the leaf sample. In brief, 5 *μ*g of extracted RNA was used for preparing cDNA (SuperScript® III, Invitrogen, USA). The cDNA (1 *μ*L) was subjected to 30 cycles in PCR machine using Taq DNA polymerase (New England Biolabs, Ipswich, MA, USA). The specific candidate gene primers *GmST1* (forward: 5′TCTAGAATGGCGTTTGTTGCAGC CATG3′; reverse: 5′GAGCTCTCATAAGGTTCGGGGATCCTTTC3′) and *GmLAX3* (forward: 5′CTGGCAGGGTTTTGCATTAT3′; reverse: 5′GCCTGTGCATTTCATAGCAA3′) along with actin primers (as a reference) were used for evaluating target gene amplification.

### 2.7. Elemental Analysis

The detailed method of Kang et al. [39] was used for elemental analysis of the plants. The lyophilized (0.5 g) crushed powder of plant samples was soaked in 0.5 M HCl and rinsed through double-distilled water before oven drying. The sample was treated with a mixture of nitric acid, sulfuric acid, and perchloric acid (10 : 1 : 4, *v/v/v*). The digested sample obtained was then analyzed by inductively coupled plasma mass spectrometry (Optima 7900DV Perkin-Elmer, Waltham, MA, USA).

### 2.8. Abscisic Acid Analysis

The method described by Kang et al. and Qi et al. [39, 55] was followed to extract and quantify ABA. Briefly, 0.5 g of powder sample was extracted with 95% isopropanol: 5% acetic acid solution; then, filtrate and standard ABA (20 ng/mL) were added to the mixture. The extracts were dried and methylated by adding diazomethane for GC/MS-SIM analysis (6890N network GC system, and 5973 network mass selective detector; Agilent Technologies, Palo Alto, CA, USA). For quantification, the Lab-Base data system software (Thermo Quest, Manchester, UK) was used to monitor responses to ions of m/e 162 and 190 for Me-ABA and of m/e 166 and 194 for Me-[2H6]-ABA.

### 2.9. Analysis of Antioxidant Enzymes

The detailed method of Marklund and Marklund [56] was adapted for the SOD activity assay. Briefly, leaf samples (100 mg) were homogenized with 0.01 M phosphate buffer at pH 7.0 and centrifuged (17,000*g*/4°C/15 min). The supernatant was used as a crude enzyme extract and passed through a reaction mixture containing Tris-HCl buffer (2 mL), pH 8.2, double-distilled water (2 mL), and 2 mM pyrogallol (0.5 mL). The absorption of the assay mixture and blank (lacking pyrogallol or tissue homogenate) was measured at 470 nm using a spectrophotometer (Shimadzu, Kyoto, Japan) at 180 s intervals. The data were expressed as units/mg of protein. To determine the reduction in glutathione concentration, each sample (500 mg) was treated with 2 mL of 10% trichloroacetic acid and centrifuged at 10,000 rpm for 15 min at 4°C. The resulting supernatant (1 mL) was combined with 0.5 mL of Ellman's reagent and 3 mL of 15 mM sodium phosphate buffer (pH 7.4) and was incubated for 5 min at 30°C. The absorbance was measured at 412 nm using a spectrophotometer [57, 58].

### 2.10. Statistical Analysis

The present study was conducted in a completely randomized design, wherein each treatment had 10 replications. All statistical analyses, including DMRT analysis, were performed using Statistic Analysis System (SAS 9.1), and for graphical presentation, GraphPad Prism software (version 5.0, San Diego, California, USA) was used.

## 3. Results

### 3.1. PGPR Bacterial Isolates Screening for Siderophore Production, Phosphate Solubilization, and Indole-3-Acetic Acid Production

Initially, we isolated a total of 126 rhizobacterial strains from four plants (*A. princeps*, *C. ficifolium*, *O. biennis*, and *E. crus-galli*). The roots of *E. crus-galli* revealed 46 rhizospheric bacterial isolates, which was the highest number among the four plant species, followed by *C. ficifolium* with 32 rhizospheric bacteria, *A. princeps* with 26 rhizospheric bacteria, and *O. biennis* with 22 rhizospheric bacteria (Supplementary [Supplementary-material supplementary-material-1]).

For the assessment of plant growth-promoting (PGP) traits, different morphological and biochemical tests were conducted. In a colorimetric assay for IAA production, a total of 39 bacterial isolates displayed positive results using Salkowski reagent. However, about 13 rhizospheric bacterial isolates revealed siderophores on CAS agar medium, whereas 14 isolates showed phosphate solubilization capability on PVK medium (Supplementary [Supplementary-material supplementary-material-1](a) and [Supplementary-material supplementary-material-1](b)).

### 3.2. Bioassay Assessment and Molecular Identification of Bacterial Isolates

Through bioassay assessment, it was found that seven isolates were capable to represent multiple PGP traits in different media. Hence, these isolates were selected, and their PGP roles were further studied through inoculation on gibberellin-deficient rice mutant *Waito-C* seedlings. A total of five selected bacterial isolates on *Waito-C* rice revealed significantly increased growth and fresher biomass than other screened isolates and control plants (Supplementary [Supplementary-material supplementary-material-1](c)). These bacterial isolates were evaluated for additional traits.

**Figure 1:**
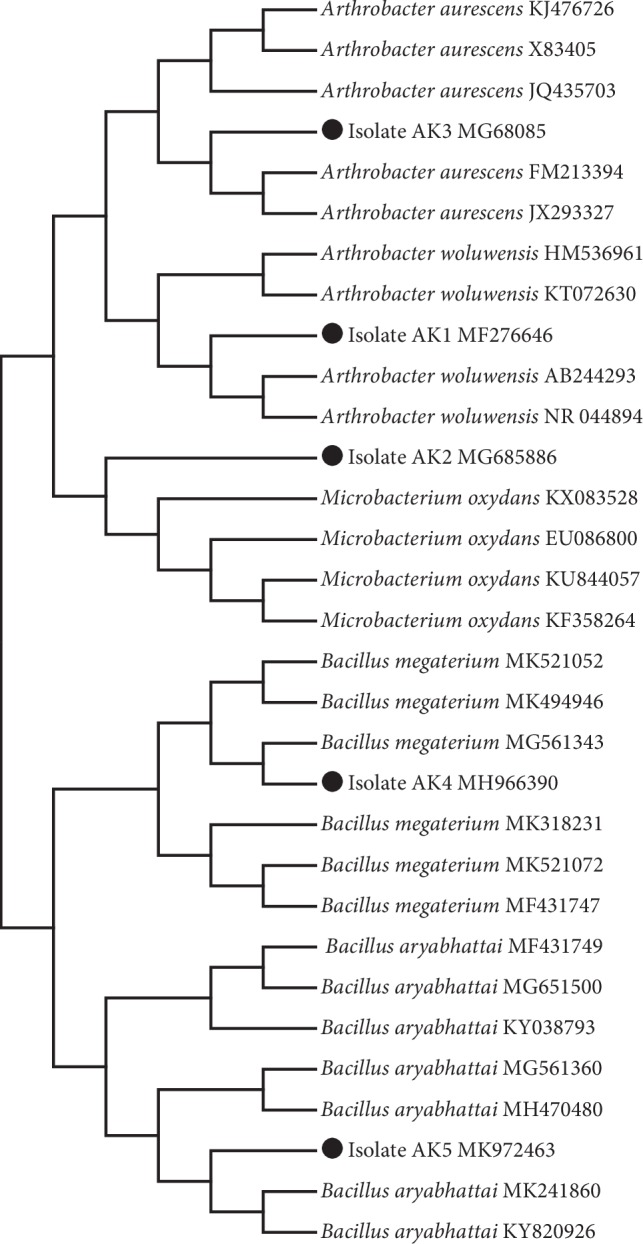
Phylogenetic analysis of rhizospheric bacterial strains isolated from the root rhizosphere of different plants *Artemisia princeps* (Korean mugwort), *Chenopodium ficifolium* (nettle-leaved goosefoot), *Oenothera biennis* (evening star), and *Echinochloa crus-galli* (cockspur grass).

For molecular identification and phylogenetic analysis of the isolates (05), the 16S rRNA genes were amplified and sequenced and compared against a database of known 16S rRNA sequences. The sequences were then submitted to NCBI to get accession numbers (Figure 1). Our analysis revealed that the rhizospheric bacteria AK1, AK2, AK3, AK4, and AK5 showed sequence identity with *Arthrobacterwoluwensis*, *Microbacterium oxydans*, *Arthrobacter aurescens*, *Bacillus megaterium*, and *Bacillus aryabhattai*, respectively. Additionally, the neighbor-joining (NJ) method was used to construct a phylogenetic tree for 16S with MEGA 6 after sequence alignment using Clustal W (version 7.222). The results revealed that AK1 and AK3 exhibited a high level of 16S rRNA sequence identity (99%) with *A. woluwensis* and *A. aurescens.* Similarly, AK2 formed a clade with *M. oxydans*, while AK4 and AK5 formed clades with *B. megaterium* and *B. aryabhattai* (Figure 1).

### 3.3. Phytohormones and Organic Acid Quantification in the Culture Broth of Selected Isolates

The culture filtrate of rhizospheric bacterial isolates was tested for quantifying phytohormones, such as IAAs and GAs, using GC/MS SIM. Interestingly, all of the selected strains were able to produce IAA in a significant amount (Table 1). The bacterial isolate of A. *woluwensis* AK1 produced the highest amount (4.87 ± 0.7 *μ*g mL^−1^) of IAA, followed by *M. oxydans* AK2 and *A. aurescens* AK3, which produced 2.78 ± 0.52 and 2.9 ± 0.72 *μ*g mL^−1^, and *B. aryabhattai* AK5 and *B. megaterium* AK4, which produced 1.08 ± 0.06 *μ*g mL^−1^ and 0.13 ± 0.7 *μ*g mL^−1^. Moreover, both active and nonactive GAs were also evaluated in the culture filtrates (Table 1). Our results revealed that GA_20_ was present in the CF culture of all isolates in a range of 1.4 ± 0.00 ng mL^−1^ to 0.04 ± 0.9 ng mL^−1^. Functionally active GAs included GA_4_ (3.14 ± 0.2 ng mL^−1^, 1.58 ± 0.8 ng mL^−1^, 1.55 ± 0.06 ng mL^−1^, and 1.4 ± 0.6 ng mL^−1^) which was detected in CF of *B. megaterium* AK4, *M. oxydans* AK2, *A*. *woluwensis* AK1, and *A. aurescens* AK3 isolates, respectively.

In this manner, GA_1_ was detected in *A. aurescens* AK3 and *B. megaterium* AK5 (0. 5 ± 0.6 ng mL^−1^and 2.5 ± 0.11 ng mL^−1^, respectively), whereas GA_7_ was detected in *M. oxydans* AK2 (0.45 ± 0.6 ng mL^−1^). Conversely, inactive types of GAs present in the CF culture of the isolates were GA_5_, GA_8_, GA_12_, GA_15_, GA_19_, GA_24_, and GA_36,_ and the highest amount of GA_12_ was detected in *M. oxydans* AK2 (7.32 ± 0.11 ng mL^−1^). Only GA_5_, GA_19_, and GA_24_ were detected in *B. aryabhattai* AK5 (0.07 ± 0.01 ng mL^−1^), *M. oxydans* AK2 (0.02 ± 0.04 ng mL^−1^), and *A. aurescens* AK3 (0.175 ± 0.8 ng mL^−1^) (Table 1).

Organic acid analysis revealed that the CF of the selected isolates produced malic acid, quinic acid, succinic acid, lactic acid, formic acid, acetic acid, butyric acid, and gallic acid (Table 2). Our results also showed that the CF of all strains contained quinic acid and acetic acid. The highest amounts of quinic acid and acetic acid were observed in *A. aurescens* AK3 (2.62 ± 0.9 ng mL^−1^) and *M. oxydans* AK2 (5.51 ± 0.9 ng mL^−1^), respectively. Only lactic acid and gallic acid were detected in the culture filtrate of *M. oxydans* AK2 (1.87 ± 0.4 ng mL^−1^) and *B. megaterium* AK4 (0.04 ± 0.1 ng mL^−1^) (Table 2). Malic acid was detected in *M. oxydans* AK2 (4.62 ± 0.8 ng mL^−1^) and *A. aurescens* AK3 (2.36 ± 0.6 ng mL^−1^). Butyric acid was detected in trace amounts in the CF of *A. woluwensis* AK3 (0.33 ± 0.5 ng mL^−1^) and in the highest amount in the CF of *M. oxydans* AK2 (2.51 ± 0.8 ng mL^−1^).

### 3.4. Ameliorative Effect of Bacterial Isolates in Alleviating Salinity Stress

**Figure 2:**
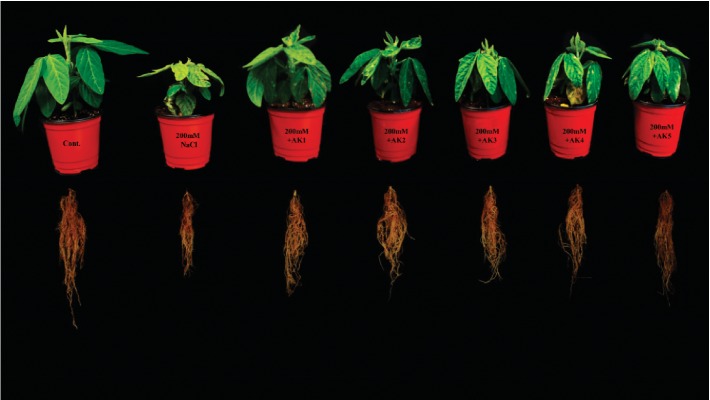
Effects of selected rhizospheric bacterial isolates on the growth attributes of soybean plants under NaCl concentrations (200 mM).

The selected rhizospheric strains were evaluated for salinity stress alleviation effects in soybean plants, which revealed some interesting results under 200 mM of NaCl stress. The bacterial isolates greatly mitigated the adverse effects of salinity stress and significantly influenced soybean growth, biomass, and chlorophyll content compared to NaCl-stressed plants (Table 3; Figure 2). In our results, a significant decrease in shoot length (30.24%) and root length (36.09%) was observed in 200 mM NaCl-stressed soybean plants compared with control-unstressed plants. However, with the combined inoculation of rhizospheric bacteria, significant increases in shoot length from 7.55% to 23.52% and root length from 11.92% to 31.17% were observed. Significant increases in shoot length were observed with *A*. *woluwensis* AK1 (23.52%) treatment and *M. oxydans* AK2 (17.89%) treatment, whereas the bacterial strains *A*. *woluwensis* AK1 and *A. aurescens* AK3 significantly increased root length up to 31.17% and 24.99%, respectively.

Similarly, fresh and dry weights were significantly higher for plants treated with bacterial isolates compared to salt-treated soybean plants. NaCl stress (200 mM) decreased the fresh shoot and root weights (70% and 69%) and dry shoot and root weights (72.45% and 71.11%, respectively) compared with control soybean plants. However, compared with salt-stressed soybean plants, plants treated with these strains showed significantly greater increases in fresh shoot weight from 15.65% to 106.39%, fresh root weight (53% to 114.49%), dry shoot weight (61.03% to 172.72%), and dry root weight (67.63% to 118.87%) (Table 3; Figure 2).

We also evaluated the effect of NaCl stress in the presence and absence of selected rhizospheric bacterial isolates on the chlorophyll content. The chlorophyll content was found to be significantly decreased in NaCl-stressed plants by up to 48.63% when compared with control plants. However, plants treated with these bacterial strains showed a prominent increase in chlorophyll content of soybean from 28.43% to 63.24% as compared to salt-treated plants (Table 3; Figure 2). Hence, the prolific effect of rhizospheric bacterial isolates mitigated the adverse effects of NaCl stress and promoted root/shoot growth and chlorophyll content.

### 3.5. Endogenous ABA Content of the Soybean Plants

**Figure 3:**
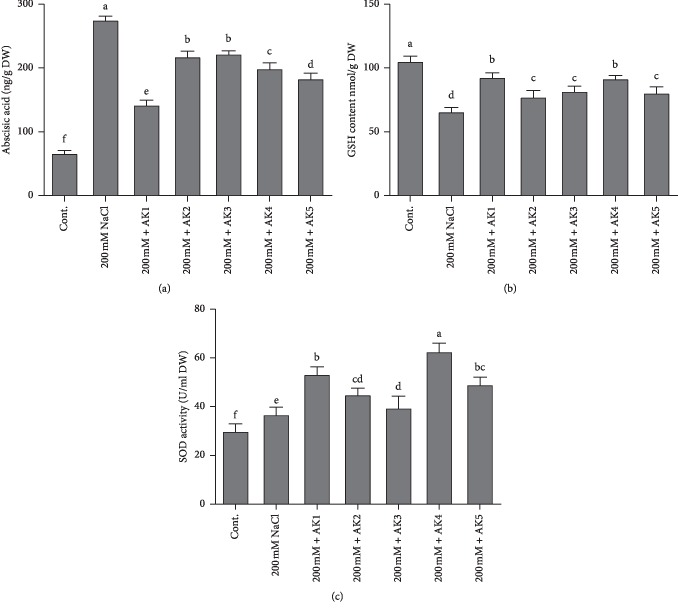
Endogenous abscisic acid (ABA), reduced glutathione (GSH), and superoxide dismutase (SOD) activity quantification in rhizospheric bacteria inoculated on soybean plants. (a) shows ABA, (b) shows the amount of GSH, and (c) shows SOD activity under normal and stressful conditions. Each data point is the mean of at least three replicates. Error bars represent standard errors. The bars represented with different letters are significantly different from each other as evaluated by DMRT.

The ABA level was significantly elevated to 275 ± 5.5 ng/g in salt-stressed plants compared with 66.5 ± 4.09 ng/g in control-unstressed soybean plants. However, the amount of endogenous ABA content under 200 mM salt stress was greatly reduced because of inoculation of rhizospheric bacteria (Figure 3(a)). The ABA level was most significantly decreased by the bacterial strain *A*. *woluwensis* AK1 (142.5 ± 7.05 ng/g), followed by *B. aryabhattai* AK5 (183.66 ± 8.50 ng/g). The fluctuation in the ABA level leads to different traits, such as the opening and closure of stomata, which affects the physiological responses of soybean and demonstrates the enhanced stress-mitigating capability of rhizospheric bacteria-treated plants, which showed a reduction in the level from 222.5 ± 10.50 ng/g to 142.5 ± 7.05 ng/g, compared with salt-stressed soybean plants, which had a level of 275 ± 5.5 ng/g (Figure 3(a)).

### 3.6. Regulation of Antioxidant Enzyme during NaCl Stress

Under normal growth conditions, salt-stressed soybean plants showed a 37.42% decrease in GSH biosynthesis compared with normal control soybean plants. However, the amount of GSH content under 150 mM salt stress greatly increased because of inoculation of selected rhizospheric bacterial strains. Compared with salt-stressed soybean plants, bacteria-inoculated plants showed a significant increase in the level of the key antioxidant GSH from 17.58% to 40.89%. Among the bacteria-inoculated soybean plants, the highest GSH content was observed in *A*. *woluwensis* AK1-inoculated plants (92.92 ± 5.20 ng/g), whereas the lowest GSH content was in *M. oxydans* AK2 (77.55 ± 6.71 ng/g) (Figure 3(b)).

Furthermore, SOD analysis results revealed an increase in SOD activity observed in soybean plants exposed to salt stress and those treated with a combined inoculation of bacterial strains compared with normal control plants. The results showed that soybean plants treated with 200 mM NaCl showed enhanced SOD activity (22.80%). However, a combined inoculation of salt and selected bacterial strains remarkably increased SOD activity (31.90% to 108.95%). Among the bacteria-inoculated soybean plants, the highest GSH content was observed in *B. megaterium* AK4 inoculated plants (108.95%), whereas the lowest GSH content was noted in *A. aurescens* AK3-inoculated plants (31.90%) (Figure 3(c)).

### 3.7. Role of Bacterial Isolates in Ion Uptake during NaCl Stress of Soybean

**Figure 4:**
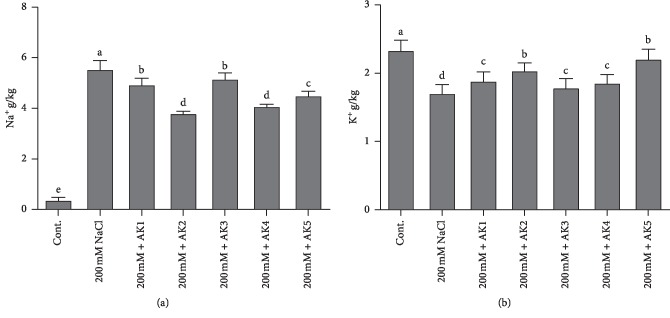
Evaluation of ICP analysis for Na^+^ and K^+^ contents in soybean shoots. (a) Na^+^ uptake and (b) K^+^ contents. Each data point is the mean of at least three replicates. Error bars represent standard errors. The bars represented with different letters are significantly different from each other as evaluated by DMRT.

ICP-MS analysis of the Na^+^ content was performed in control and salt-treated plants with combined inoculation of rhizospheric-inoculated soybean plants. The results showed that NaCl stress (200 mM) increased the Na^+^ content in soybean plants. However, soybean plants inoculated with bacterial strains significantly decreased the Na^+^ content to a range of 6.83%–31% (Figure 3(a)). The highest reduction in the Na^+^ content was to 6.83%, observed in plants inoculated with *A. aurescence* AK3, whereas the least reduction was to 31%, observed in plants inoculated with *M. oxydans* AK2, compared with 200 mM NaCl-stressed soybean plants (Figure 4(a)).

ICP analysis of K^+^ showed a significant decrease in K^+^ concentrations (24.78%) in soybean plants under 200 mM NaCl stress compared with control plants. However, the K^+^ content under 200 mM NaCl stress greatly increased because of bacterial inoculation. The bacterial strain *B. aryabhattai* AK5 significantly increased K^+^ content (25.56%) followed by *M. oxydans* AK2 (15.92%). Among bacteria-inoculated plants, the lowest K^+^ concentration was observed in *A. aurescence* AK3 (1.76%)-inoculated soybean plants compared with 200 mM NaCl-stressed plants (Figure 4(b)).

### 3.8. Gene Expression during NaCl Stress of Soybean Plants

**Figure 5:**
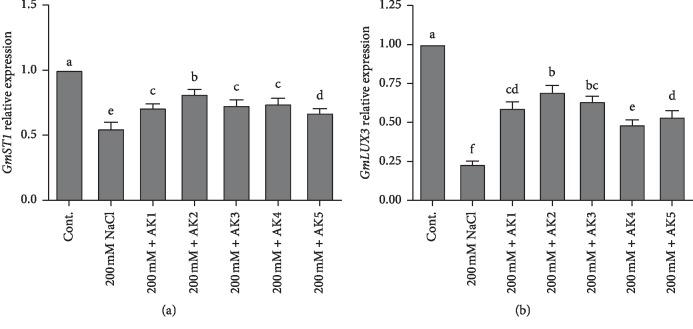
Relative expression of *GmST1* and *GmLUX3* genes in soybean plants. The values were calculated relative to those of actin gene expression and are means of three replicates. Error bars represent standard errors. The bars represented with different letters are significantly different from each other as evaluated by DMRT analysis.

*GmST1* is a soybean salt tolerance gene that functions in salt stress tolerance, and its protein product functions to decrease the production of ROS. Thus, we investigated the expression and regulation of *GmST1* under high NaCl stress (200 mM) with and without the presence of selected rhizospheric strains inoculated to soybean plants using qRT-PCR. Our results revealed that salt stress significantly decreased the expression of *GmST1* up to 44.99% compared with unstressed control soybean plants. However, salt stress with combined inoculation of rhizospheric bacteria augmented the expression of *GmST1* up to 18.99% to 32.99% (Figure 5(a)). Compared with NaCl-stressed soybean, the highest expression of *GmST1* was observed in *M. oxydans* AK2 (18.99%)-inoculated soybean plants while lowest expression was observed in *B. aryabhattai* AK5 (32.99%)-inoculated soybean plants.

Similarly, *GmLAX3* plays an important role and may be the most promising candidate gene for improving soybean adaptability against salinity stress. The expression of *GmLAX3* in NaCl-stressed plants decreased 76.66% compared with control soybean plants. Our results revealed that the upregulation of *GmLAX3* gene significantly increased soybean resistance to NaCl stress plant inoculated with selected rhizospheric strain. The expression level of *GmLAX3* increased from 30.33% to 51.33% in soybean plants with combined inoculation of bacterial strains compared with NaCl stress (200 mM) plants. Furthermore, the highest expression of *GmLAX3* was observed in *M. oxydans* AK2 (43.13%)-inoculated plants, whereas the lowest expression was in *B. megaterium* AK4-inoculated plants and *B. aryabhattai* AK5-inoculated plants (16.6% and 17.30%), respectively (Figure 5(b)).

## 4. Discussion

Salinity adversely affects the plant growth by exerting osmotic ionic stresses, primarily due to elevated Na^+^ levels in the soil, which drive out the water from the plant cell, thus affecting the turgor pressure, leaf area, chlorophyll metabolism, and photosynthetic activity [59]. Since salt stress-resistant PGPR are recognized as potential stress relievers in plants due to their ability to reduce the Na^+^/K^+^ ratio. In the present study, the application of *Arthrobacter woluwensis* (AK1), *Microbacterium oxydans* (AK2), *Arthrobacter aurescens* (AK3), *Bacillus megaterium* (AK4), and *Bacillus aryabhattai* (AK5) significantly enhanced the K^+^/Na^+^ ratio in plants and enhanced the PGP characteristics, including photosynthetic activity (Table 3). NaCl stress exposure (200 mM) significantly reduced shoot and root lengths and fresh and dry weights of soybean plants compared to controls (unstressed plants). Similarly, previous reports showed that NaCl stress affected the growth of sweet sorghum [40], groundnut [12], okra [41], pepper [42], wheat [43], and soybean [39, 60]. The selected rhizospheric strains greatly mitigated the adverse effects of salinity stress and significantly enhanced soybean growth, biomass, and chlorophyll content compared with NaCl-stressed plants (Table 3; Figure 2). Recently, plant growth-promoting rhizospheric bacteria have been used to alleviate salt stress and improve crop production [25, 39, 58, 61–66]. Thus, here we describe how the findings of our results provide an insight on advancing the strategy to counter salinity stress.

The interaction of plants and microbes occurs in the rhizosphere due to rhizo-deposition, which includes several chemical compounds exudated from the plant root, such as organic acids and phytohormones [67]. Organic acids are also considered an important source of carbon and rich in energy. Our results revealed that cultured filtrates of selected isolates produced malic acid, quinic acid, succinic acid, lactic acid, formic acid, acetic acid, butyric acid, and gallic acid (Table 2). Previous reports showed that different bacteria produce various types of organic acids [25, 46, 66, 68]. In addition, organic acid enhances the degree and rate of metal dissolution, increases pH homeostasis, and promotes plant growth [69]. Eventually, these aspects influence chemical signals between root and microbes and promote the microbial community and support its functional role in plants [70, 71]. Furthermore, the microbes that produce organic acids have an important role in the solubilization of mineral substances. One of the most accepted examples is the mineralization of phosphate [72]. Phosphate-solubilizing PGPR reportedly produce organic acids that facilitate the uptake of P as well as essential nutrients from the soil [73]. The microbes used in the current experiment have an innate ability to produce organic acids (Table 2) and enhance phosphate solubilizing activity (Supplementary [Supplementary-material supplementary-material-1]). These microbial strategies may have influenced the growth and development of the plant. Similar results have been reported by several authors where organic acid and phosphate solubilizing bacteria mitigated abiotic stress by regulating phytohormones and antioxidants [25, 39, 46, 66, 68]. The combination of IAA production ability, phosphorous solubilization, and siderophore production of rhizospheric bacteria is greatly beneficial to plant rhizospheric soil as it mitigates the adverse effects of salinity stress [42, 74, 75].

The microbes used in the current study actively participated in IAA, GA, OA (organic acid), and siderophore production and phosphate solubilization. The application of bacterial inoculants to plant roots is expected to contain sufficient amount of growth regulators to influence future plant growth and development [76, 77]. There is the possibility that more hormones and other bioactive secondary metabolites are readily available to the plant for absorption and contribution in the root growth, cell elongation, tissue differentiation, and plant growth. It has been reported that the IAA produced by rhizosphere bacteria will increase the length and root surface of plants, thus offering them better access to nutrients available in the soil [78]. Similarly, various researchers revealed that IAA-producing bacteria significantly enhance plant growth under saline stress conditions [79, 80]. In connection to this, our bacterial isolates also produced IAA and greatly mitigated the adverse effects of NaCl stress on soybean plants (Table 1). Similarly, microorganisms that produced GAs play a key role in plant growth promotion and mitigating salt stress [39, 66]. Our selected bacterial isolates produced different bioactive and nonbioactive GAs (Table 1) and mitigated salinity stress in soybean plants. Many previous reports have shown the ameliorative effects of GAs on plant growth under abiotic stress [25, 39, 66]. It is considered that although there are several forms of GA, biologically active forms are limited to GA_1_, GA_3_, and GA_4_ [46]. These biologically active GA forms promote plant growth by reducing stress hormones like ABA [81]. As plants suspect stress, they regulate stress hormones like ABA through active chemical signals, which lead to increased sensitivity of plants for stomatal conductance [82]. As microbial interaction mitigates the stress effects by reducing the ABA content [83], similar results were observed in our study where selected rhizospheric bacterial inoculates resulted in decreased ABA content and increased plant growth parameters (Table 3; Figures 2 and 3(a)). Similarly, salinity stress leads to the formation of ROS, which cause cellular toxicity and damage to cell structures in plants [84, 85]. However, antioxidant enzymes, such as superoxide dismutase (SOD), peroxidase, glutathione reductase, and catalase, which protect the plant against cellular stress, remove free radicals and scavenge excess ROS. In the present study, the rhizospheric bacteria-inoculated plants revealed increases in SOD and GSH contents **(**Figures 3(b) and 3(c)). Similar results were reported for lettuce, potato, and okra [41, 86], wherein PGPB enhanced the activities of different ROS-scavenging enzymes under increasing salinity stress.

When microbes are introduced in the roots, they alter toxic ion uptake and modify the physical barriers around the rhizosphere by the formation of extensive rhizosheaths through the production of exopolysaccharides, reduction of foliar accumulation of toxic ions, and improvements in the nutritional status of both macro and micronutrients [30]. The greater accessibility of plants to nutrients is particularly due to changes in rhizospheric pH due to organic acid secretion and chelation through siderophore production [87]. This leads to the maintenance of water homeostasis and osmolyte accumulation and stimulates carbohydrate metabolism and transport to maintain source-sink relations that avoid photo-inhibition during the osmotic state of salinity; furthermore, this reduces Na^+^ toxicity and ultimately promotes plant growth [30]. Salt stress causes higher accumulation of Na^+^, which competes with K^+^-binding proteins and decreases protein synthesis. Na^+^ expulsion and K^+^ influx is the most important plant strategy for relieving salinity stress [88]. The present results showed that salt stress-inoculated soybean plants had higher Na^+^ and decreased K^+^ levels (Figure 4). However, the inoculation of selected rhizospheric strains resulted in decreased Na^+^ and increased K^+^ concentrations (Figure 4). Similarly, it was reported that Na^+^ expulsion and K^+^ influx can be restricted to roots of various plants (maize and soybean) using different bacterial strains [43, 89, 90].

The identification of salt tolerance genes is of great importance to develop sustainable agriculture practices. Several crop plants can be genetically engineered for agricultural practice in a salt-stressed environment. Furthermore, genome-wide transcriptomic analysis in soybean revealed that a number of hormone-related genes were differentially expressed in shoots and roots under salinity and drought stresses [91]. The candidate genes, such as *GmLAX3* and *GmST1*, for cation antiporters and salt tolerance have been identified in soybean. *GmST1* gene reportedly reduces the toxic effect of ROS production and enhances ABA sensitivity. *GmLAX3* has a similar role in the context of IAA regulation. Results from the present study showed that salinity stress down-regulated the expression of *GmST1* (Figure 5(a)). However, the inoculation of rhizospheric bacterial strains stimulated the expression of *GmST1* in soybean plants exposed to salt stress (Figure 5(a)). The expression of *GmST1* was regulated through an ABA-dependent pathway and decreased production of ROS during salt stress. Similar results of *GmST1* overexpression showed strong tolerance in *Arabidopsis* to salinity stress [20], making it a potential candidate gene for genetic engineering of salt-tolerant plants. Furthermore, *GmLAX3* is among the key genes involved in salt stress response in plants [24, 25]; it encodes a multimembrane spanning transmembrane protein and functions in auxin uptake and intercellular auxin flow [24]. The present results revealed that *GmLAX3* expression was down-regulated under salinity stress. However, rhizospheric bacteria stimulated the expression of *GmLAX3* in soybean plants exposed to salt stress (Figure 5(b)). As reported previously, the overexpression of *GmLAX3* enhanced salt stress tolerance of plants [24, 25, 92].

## 5. Conclusion

The use of PGPR could be an efficient way to confer salinity stress resistance in crop plants. Presently, the ameliorative role of PGPR was evaluated in soybean plants under salt stress conditions. The experimental data revealed that salt stress-resistant PGPR—*A. woluwensis* (AK1), *M. oxydans* (AK2), *A. aurescens* (AK3), *B. megaterium* (AK4), and *B. aryabhattai* (AK5)—greatly helped in the recovery of soybean plants by producing bioactive metabolites which activated antioxidants (GSH and SOD), modulated phytohormones (ABA), maintained osmotic balance by suppressing Na^+^ and promoting K^+^ ion uptake, and regulated salt tolerance (*GmST1*) and IAA-mediating (*GmLAX3*) genes. Hence, the present research supports and takes further the notion of using halotolerant PGPR to develop eco-friendly biofertilizers for enhanced growth and quality yield of crop plants grown under salinity stress.

## Figures and Tables

**Table 1:** Quantification of IAA and gibberellins produced by rhizospheric bacterial isolates.

	IAA	GA_1_	GA_4_	GA_5_	GA_7_	GA_8_	GA_12_	GA_15_	GA_19_	GA_20_	GA_24_	GA_36_
*A. woluwensis* AK1	4.87 ± 0.73^A^	ND	1.55 ± 0.06^A^	ND	ND	0.045 ± 0.04^D^	0.755 ± 0.08^B^	ND	ND	0.393 ± 0.03^C^	ND	ND
*M. oxydans* AK2	2.78 ± 0.52^B^	ND	1.58 ± 0.8^B^	ND	0.455 ± 0.6^C^	0.05 ± 0.04^D^	7.415 ± 0.1^A^	0.265 ± 0.08^C^	0.02 ± 0.04^D^	0.485 ± 0.09^C^	ND	0.36 ± 0.09^C^
*A. aurescens* AK3	2.69 ± 0.72^B^	0.52 ± 0.6^D^	1.4 ± 0.1^C^	ND	ND	8.32 ± 0.11^A^	0.43 ± 0.09^DE^	ND	0.401 ± 0.9^DE^	0.12 ± 0.8^E^	ND	ND
*B. megaterium* AK4	0.13 ± 0.03^D^	ND	3.147 ± 0.2^A^	ND	ND	0.27 ± 0.09^A^	ND	ND	ND	1.4 ± 0.03^C^	ND	0.385 ± 0.04^B^
*B. aryabhattai* AK5	1.08 ± 0.1^B^	2.5 ± 0.21^A^	ND	0.07 ± 0.01^C^	ND	0.08 ± 0.07^C^	ND	ND	ND	0.04 ± 0.01^D^	ND	ND

Each data point is the mean of at least three replicates. Error bars represent standard errors. The bars represented with different letters are significantly different from each other as evaluated by DMRT.

**Table 2:** Types and quantity of organic acids in the culture broth of rhizospheric bacteria.

	Malic acid	Quinic acid	Succinic acid	Lactic acid	Acetic acid	Butyric acid	Gallic acid
*Arthrobacter woluwensis* AK1	ND	1.53 ± 0.7^AB^	2.58 ± 0.7^A^	ND	2.38 ± 0.8^A^	0.33 ± 0.5^B^	ND
*Microbacterium oxydans* AK2	4.62 ± 0.8^A^	1.58 ± 0.6^C^	2.75 ± 0.8^B^	1.87 ± 0.4^C^	5.51 ± 0.9^A^	2.51 ± 0.8^B^	ND
*Arthrobacter aurescens* AK3	2.36 ± 0.6^A^	2.62 ± 0.9^A^	0.94 ± 0.7^B^	ND	0.61 ± 0.5^B^	ND	ND
*Bacillus megaterium* AK4	ND	0.05 ± 01^B^	ND	ND	1.64 ± 02^A^	ND	0.04 ± 01^B^
*Bacillus aryabhattai* AK5	ND	0.06 ± 01^B^	ND	ND	0.31 ± 02^A^	ND	ND

Each data point is the mean of at least three replicates. Error bars represent standard errors. The bars represented with different letters are significantly different from each other as evaluated by DMRT.

**Table 3:** Influence of rhizospheric bacteria on growth attributes and chlorophyll content of soybean plants grown with/without NaCl.

	Shoot length (cm)	Root length (cm)	Shoot fresh weight (mg)	Root fresh weight (mg)	Shoot dry weight (mg)	Root dry weight (mg)	SPAD (C.C)
Control	139.73 ± 3.60^A^	251.00 ± 3.60^A^	12.56 ± 0.65^A^	7.66 ± 0.55^A^	1.86 ± 0.03^A^	0.60 ± 0.10^A^	46.83 ± 3.61^A^
NaCl (200 mM)	97.46 ± 1.85^F^	160.40 ± 3.41^F^	3.74 ± 0.35^E^	2.30 ± 0.22^D^	0.51 ± 0.01^F^	0.17 ± 0.03^C^	24.03 ± 3.10^D^
*Arthrobacter woluwensis* AK1	120.40 ± 2.62^B^	210.40 ± 3.41^B^	7.73 ± 0.50^B^	4.60 ± 0.31^B^	1.40 ± 0.11^B^	0.37 ± 0.03^B^	39.23 ± 2.54^B^
*Microbacterium oxydans* AK2	114.9 ± 2.15^C^	195.23 ± 2.15^C^	6.13 ± 0.41^C^	4.93 ± 0.25^B^	1.10 ± 0.10^C^	0.30 ± 0.02^B^	35.70 ± 3.05^BC^
*Arthrobacter aurescens* AK3	112.03 ± 1.95^CD^	200.50 ± 2.68^C^	5.43 ± 0.35^CD^	3.63 ± 0.31^C^	0.82 ± 0.06^E^	0.37 ± 0.22^B^	35.63 ± 3.55^BC^
*Bacillus megaterium* AK4	104.83 ± 1.55^E^	179.53 ± 3.32^E^	5.23 ± 0.35^D^	3.53 ± 0.30^C^	0.98 ± 0.04^D^	0.29 ± 0.20^B^	32.06 ± 3.51^C^
*Bacillus aryabhattai* AK5	110.56 ± 1.53^D^	185.30 ± 2.26^D^	4.33 ± 0.25^E^	3.73 ± 0.25^C^	0.83 ± 0.04^E^	0.29 ± 0.03^B^	30.86 ± 3.12^C^

Each data point represents the mean of three replicates ± standard errors. The different letters represent data points that are significantly different from each other as evaluated by DMRT analysis.

## Data Availability

All data generated or analyzed during this study are included in this article.

## Abbreviations

PGPR: Plant growth rhizospheric bacteria
IAA: Indole-3-acetic acid
GA: Gibberellin
ROS: Reactive oxygen species
SOD: Superoxide dismutase
GSH: Reduced glutathione (GSH)
*GmLAX*: Auxin resistant 1
*GmST1*: Soybean salt tolerance 1
PGP: Plant growth-promoting
GC/MS SIM: Gas chromatography-mass spectrometry under selected ion monitoring mode
HPLC: High-performance liquid chromatography
ICP: Inductively coupled plasma
CF: Culture filtrate.

## Supplementary Materials

Supplementary Materials Table S1: description of plant species, rhizospheric bacteria isolation, and the number of yielded isolates with individual/multiple plant growth-promoting characteristics. Figure S1 Siderophores and phosphate solubilization activity on PVK and CAS medium are shown. (a) Capability of siderophores production, (b) Phosphate solubilization activity, and (c) Growth promotion of Waito-C rice using rhizospheric bacteria.
