# Supplementary material for: Halotolerant Rhizobacterial Strains Mitigate the Adverse Effects of NaCl Stress in Soybean Seedlings
Source: Biomed Res Int. 2019 Oct 20;2019:9530963. doi: 10.1155/2019/9530963 (PMC6925695; doi:10.1155/2019/9530963)
Supplement: Supplementary Materials — Table S1: description of plant species, rhizospheric bacteria isolation, and the number of yielded isolates with individual/multiple plant growth-promoting characteristics. Figure S1 Siderophores and phosphate solubilization activity on PVK and CAS medium are shown. (a) Capability of siderophores production, (b) Phosphate solubilization activity, and (c) Growth promotion of Waito-C rice using rhizospheric bacteria. [file 9530963.f1.zip › 9530963.f1/Supplementary figure 1.pptx]

## Slide 1
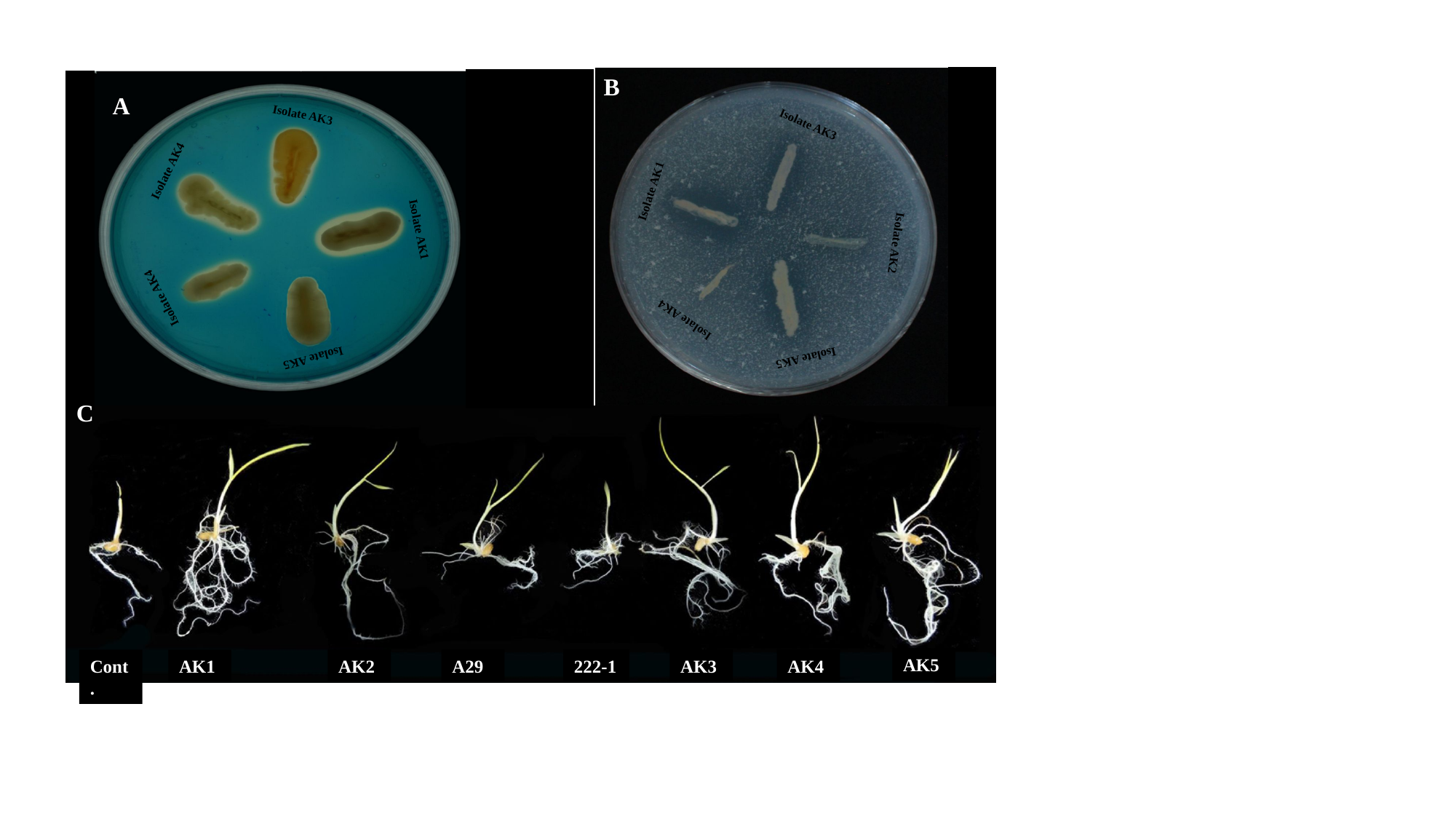

B
Isolate AK3
Isolate AK1
Isolate AK2
Isolate AK4
Isolate AK5
A
Isolate AK3
Isolate AK4
Isolate AK1
Isolate AK4
Isolate AK5
C
AK5
AK2
A29
222-1
AK3
AK4
Cont.
AK1
