# Supplementary material for: Halotolerant Rhizobacterial Strains Mitigate the Adverse Effects of NaCl Stress in Soybean Seedlings
Source: Biomed Res Int. 2019 Oct 20;2019:9530963. doi: 10.1155/2019/9530963 (PMC6925695; doi:10.1155/2019/9530963)
Supplement: Supplementary Materials — Table S1: description of plant species, rhizospheric bacteria isolation, and the number of yielded isolates with individual/multiple plant growth-promoting characteristics. Figure S1 Siderophores and phosphate solubilization activity on PVK and CAS medium are shown. (a) Capability of siderophores production, (b) Phosphate solubilization activity, and (c) Growth promotion of Waito-C rice using rhizospheric bacteria. [file 9530963.f1.zip › 9530963.f1/Supplementary Table 1.docx]

**Table S 1.**

Description of plant species, rhizospheric bacteria isolation, and the number of yielded isolates with individual/multiple plant growth-promoting characteristics

| **Plants Name** | **No of isolates** | **Isolates having single plant growth promoting characteristics** | | | **Isolates with multiple PGP characteristics** |
| --- | --- | --- | --- | --- | --- |
|  |  | **IAA production** | **Siderophore** | **Phosphate** |  |
| **Rhizospheric isolates** | | | | | |
| *Artemisia princeps* Pamp. | 26 | 11 | 2 | 4 | 2 |
| *Chenopodium ficifolium* Smith | 32 | 12 | 4 | 1 | 1 |
| *Oenothera biennis* L. | 22 | 5 | 2 | 0 | 0 |
| *Echinochloa crus-galli* (L.) Beauv. | 46 | 11 | 5 | 9 | 4 |
